# Dietary Patterns of Infants and Toddlers Are Associated with Nutrient Intakes

**DOI:** 10.3390/nu4080935

**Published:** 2012-08-13

**Authors:** Lisa G. Smithers, Rebecca K. Golley, Laima Brazionis, Pauline Emmett, Kate Northstone, John W. Lynch

**Affiliations:** 1 Discipline of Public Health, School of Population Health, University of Adelaide, Mail Drop DX650550, Adelaide 5005, South Australia, Australia; Email: laimab@unimelb.edu.au (L.B.); john.lynch@adelaide.edu.au (J.W.L.); 2 Sansom Institute for Health Research, School of Health Sciences, University of South Australia, Adelaide 5000, South Australia, Australia; Email: rebecca.golley@unisa.edu.au; 3 School of Social and Community Medicine, University of Bristol, Bristol BS82BN, UK; Email: P.M.Emmett@bristol.ac.uk (P.E.); Kate.Northstone@bristol.ac.uk (K.N.)

**Keywords:** dietary patterns, infants, toddlers, nutrient intake, ALSPAC

## Abstract

Dietary patterns are a useful summary measure of diet. Few studies have examined the nutrient profiles underpinning the dietary patterns of young children. The study aim is to determine whether dietary patterns at 6 and 15 months of age are associated with nutrient intakes at 8 and 18 months, respectively. Participants were children from the Avon Longitudinal Study of Parents and Children who had complete dietary pattern and nutrient intake data (*n* = 725 at 6–8 months, *n* = 535 at 15–18 months). The association between tertiles of dietary pattern scores and nutrient intake was examined using a non-parametric test for trend. Scores on the *home-made traditional* pattern (6–8 months) were positively associated with median energy intake. Each dietary pattern had different associations with energy-adjusted intakes of macro- and micro-nutrients. At both times, the *discretionary* pattern was positively and the *ready-prepared baby foods* pattern was negatively associated with sodium intake. At 6–8 months, calcium and iron intakes decreased across scores on the *home-made traditional* and *breastfeeding* patterns, but increased across the *ready-prepared baby food* patterns. These findings highlight that dietary patterns in infants and toddlers vary in their underlying energy and nutrient composition.

## 1. Introduction

Over the last two decades it has become increasingly acknowledged that events occurring in early life have a lasting effect on an individuals’ health [1]. The diet of infants and toddlers has short and long-term implications for health and development [2,3,4]. In the first two years of life, an infant’s diet changes from milk as the sole food source, to foods and beverages that reflect the family diet. This transition represents the most rapid change in diet over the life course and is the developmental period when dietary preferences and habits are first established [5]. It is important to be able to characterize early life dietary patterns and understand the composition of such patterns in order to examine how early diet influences later outcomes.

Principal component analysis (PCA) is a technique for characterizing the whole diet. PCA is based on correlations between foods and is used to identify foods that are frequently consumed together. Individuals are assigned a score on each dietary pattern and higher scores indicate a greater intake of the foods associated with that pattern.

A number of studies have characterized dietary patterns in infancy and toddlerhood [6,7,8,9,10,11]. For example, using dietary questionnaires at 6 and 15 months of age, we identified four dietary patterns among a United Kingdom birth cohort [7]. A *traditional* stylepattern characterized by home-prepared meats, vegetables and desserts; a*ready-prepared baby foods* pattern; and a *discretionary* pattern characterized by foods such as biscuits, sweets and crisps were identified at both ages. At 6 months, the fourth pattern was characterized predominantly by *breastfeeding* and at 15 months, by *contemporary-style* foods including herbs, legumes, nuts, raw fruit and vegetables. Similar early dietary patterns have been reported in other studies, including in a more contemporary UK birth cohort [8] and other countries including Norway [6], the Netherlands [11] and Japan [9].

While dietary patterns provide a useful summary measure of diet, it is important to understand the energy and nutrient intake underlying the pattern. However this is rarely examined, as we have been able to identify only two studies involving young children that report associations between nutrients and dietary patterns [11,12]. In 14-month-old toddlers from the Netherlands (*n* = 2420), weak correlations were observed between *health-conscious* or *Western-like* dietary patterns and energy and macronutrient intakes. The *health-conscious* pattern was positively correlated with energy, protein and polysaccharide intake, and was inversely correlated with saturated fat intake. The *Western-like* pattern was positively correlated with energy and saturated fat intake and inversely correlated with protein and polysaccharide intake. Shin *et al.* found that the total energy intake of five-year-old Korean children was associated with higher scores on each of the three patterns they identified (*Korean healthy*; *animal foods*; *sweets* pattern*s*, *n* = 1441). Energy-adjusted intake of protein, fiber, iron, calcium, vitamin A, carotene and vitamin C was associated with higher scores on the *Korean-healthy* dietary pattern. In contrast, scores on the *sweets* pattern was inversely associated with nutrient intakes. 

Understanding the energy and nutrient intakes that underpin a dietary pattern provides a measure of the patterns’ validity and can improve our understanding of the possible pathways through which diet might influence health and development. Therefore the aim of this study is to determine whether dietary patterns obtained from questionnaires at 6 and 15 months of age are associated with nutrient intakes assessed by diet record at 8 and 18 months, respectively, using data from the Avon Longitudinal Study of Parents and Children (ALSPAC). 

## 2. Experimental Section

ALSPAC is an ongoing longitudinal birth cohort study that is designed to investigate the determinants of health and development [13]. All pregnant women residing in the former county of Avon, Southwest England who were expected to deliver between 1st April 1991 and 31st December 1992 were invited to participate in ALSPAC. Information about the ALSPAC cohort and study questionnaires are published on the ALSPAC website. The study sample is considered to be broadly representative of the population although ethnic minorities, single parents and unmarried couples are slightly underrepresented compared with the 1991 National Census of women residing in Avon. Ethical approval for the study was obtained from the ALSPAC Ethics and Law Committee and the Local Research Ethics Committees. The core ALSPAC sample consists of 14,541 pregnancies and of these pregnancies, 13,988 infants survived to 1 year of age. Children in Focus (CIF) is a subsample of 1432 children born in the last 6 months of the cohort recruitment (July to December 1992) that were invited for regular clinical assessments from birth. The study described in this paper involves the 10% CIF subsample of children, whose nutrient intakes were collected at 8 and/or 18 months of age using an un-weighed 3-day diet diary [14,15], and for whom dietary patterns were extracted from dietary questionnaires at 6 and/or 15 months of age, respectively [7].

### 2.1. ALSPAC Dietary Data

#### 2.1.1. Dietary Patterns at 6 and 15 Months of Age

We have previously described the procedure for determining dietary patterns in the ALSPAC cohort at 6 and 15 months of age [7]. Briefly, dietary questionnaires were posted to the primary caregiver when the study child was 6 and 15 months old. The questionnaire listed 43 food and beverage items at 6 months, increasing to 70 items at 15 months in accordance with the wider exposure to foods at older ages. The foods and beverages listed in the questionnaires were determined by an experienced dietician (PE) and were based on foods commonly fed to infants. The questionnaires included information on breastfeeding, formula and other milks, whether foods were prepared in the home or were purchased ready-prepared [16]. An estimate of current dietary intake was obtained by asking caregivers whether their child had ever been fed a food item and how many times per week they fed their child a food item nowadays. Participants who had never consumed a food item were assigned an intake of zero. Dietary patterns were extracted using PCA with Oblimin rotation due to the greater flexibility in examining latent structures (PAWS statistical software Version 17.0, SPSS Inc., Chicago, IL, USA) [17,18]. Food and beverage data were entered into PCA as a frequency of consumption (times per week). The number of dietary patterns extracted was based on a break in the Scree plot and interpretability of the patterns. Pattern scores were calculated for each participant based on an algorithm that includes the frequency of intake and the factor loading for each food item [19,20]. Each pattern had a mean equal to zero and standard deviation equal to one. Four dietary patterns were extracted at each age and three of these patterns were similar at both 6 and 15 months of age. The first pattern common to both ages was characterized by home-prepared meat, vegetables and desserts (named the “*home-made traditional*” pattern). The second pattern was characterized by discretionary foods such as biscuits, sweets and crisps (“*discretionary*” pattern), and the third pattern was characterized by and named “*ready-prepared* *baby foods*”. At 6 months of age, a fourth pattern was characterized by breastfeeding (“*breastfeeding*”) and at 15 months, by contemporary-style foods including herbs, legumes, nuts, cheese, raw fruit and vegetables (“*home-made contemporary*”). 

#### 2.1.2. Nutrient Intake at 8 and 18 Months of Age

As described previously, nutrient intakes have been derived from an un-weighed 3-day diet diary [14,15]. In addition to the dietary questionnaires from the whole cohort at 6 and 15 months, diet diaries were collected from the CIF subsample at slightly older ages (8 and 18 months). The time lag was considered necessary to ameliorate participant burden. In the week prior to their clinic visit, caregivers were sent three 1-day diet diaries and asked to record all of the food and beverages consumed by the study child on two weekdays and one day on a weekend. The days for recording dietary data were chosen by the caregiver and were not necessarily consecutive. At the clinic, the diet diaries were reviewed by a trained researcher, with the aim of ensuring accuracy in the types of foods and drinks recorded in the diary. Any anomalies were clarified with the caregiver. Caregivers were asked to record portion sizes in household measures. Mothers of breastfed infants were asked to record the duration of each feed. A breast milk transfer rate of 10 mL per minute was assumed up to a maximum volume of 100 mL per feed [21]. This method was used to allow a direct comparison with a national British survey of 6–9 month old infants [22]. Portion sizes were transformed to weights using published food portion sizes. All foods and beverages were coded by researchers and the weights and codes were entered into the Microdiet software package (University of Salford) at 8 months and the more up-to-date and efficient Dido software (MRC Human Nutrition Resource Centre, Cambridge) at 18 months of age [15]. The average daily nutrient intakes were estimated using McCance and Widdowson’s 5th edition and supplements [23,24,25], with any additional nutrient information obtained from food manufacturers. Quality was maintained by checking and correcting the output against all the original diaries and rechecking outliers for a range of nutrients [14]. 

### 2.2. Descriptive Characteristics

Information on maternal age, education, social class, financial difficulties, marital status, tobacco smoking and number of children (<16 years) living in the family home was collected by postal questionnaires between 8 and 32 weeks gestation. Maternal education was reported in five categories ranging from a Certificate of Secondary Education (CSE), Vocational training, O(ordinary)-level (taken by the top 25% of CSE at 16 years), A(advanced)-level (involving 2 years of study beyond O-level) to Degree or higher. The CSE, O-levels and A-levels are completed at secondary school. Social class was categorized according to maternal occupation during pregnancy using standard UK classifications of occupation in six categories from class I (highest), II, III non-manual, III manual, IV and V (lowest) [26]. Financial difficulty was determined from five questions in which the caregiver reported their difficulty in affording food, clothing, heating, rent/mortgage and things needed for the baby. Caregivers were asked to respond to each question on a 4-point Likert scale that ranged from 4 (very difficult), 3 (slightly difficult), 2 (fairly difficult) to 1 (not difficult). Scores on each question were summed to give a total financial difficulties score that ranged from 5 (no difficulties) to 20 (maximum financial difficulties on all 5 questions). The top 10% of scores reflected families who were experiencing financial difficulties and included all participants with total scores ≥11. Marital status was categorized as: partnered (first or subsequent marriage), or single (divorced, widowed, separated or never married). Tobacco smoking was categorized as: never smoked, quit smoking or smoked during pregnancy. Infant sex and singleton/multiple birth information was collected by ALSPAC staff at birth, from medical records or from the infants’ birth notification.

### 2.3. Statistical Methods

A non-parametric test for trend was used to examine whether; (1) dietary pattern scores at 6 months of age were associated with nutrient intakes at 8 months, or (2) dietary pattern scores at 15 months were associated with nutrient intakes at 18 months [27]. All available data were used in each analysis such that participants may be included in both analyses, only the 6–8-month analysis or only the 15–18-month analysis. Nutrient intakes were adjusted for total dietary energy intake using the residual method [28]. Dietary pattern scores were divided into tertiles for ease of presentation; similar associations were observed if the pattern scores were divided into quintiles (data not shown). Tertile 1 has the lowest and tertile 3 has the highest scores on each dietary pattern. As described previously, the *home-made traditional* pattern at 15 months had strong negative loadings for key foods [7], which mean that lower scores are associated with higher intakes of home-made potatoes, meat products, cooked vegetables and puddings. For ease of interpretation and consistency with the other patterns we reversed the direction of the tertiles for the *home-made traditional* pattern at 15 months such that individuals with higher consumption of meat, vegetables and puddings appear in tertile 3. As some nutrient intakes are skewed, the median intakes are shown for each tertile of dietary pattern score. As we investigated 30 nutrients and 4 dietary patterns, a Bonferroni-adjusted *p*-value of <0.0005 was applied to the associations between dietary patterns and nutrient intakes. Characteristics of the sample at 6–8 months or 15–18 months were compared against the rest of the cohort using Chi-squared tests for categorical variables and independent *t*-tests for continuous variables. Statistical analyses were conducted using STATA (Intercooled 11.0, StataCorp TX).

## 3. Results

Dietary pattern scores at 6 months and nutrient intakes at 8 months were available from *n* = 725 participants. Dietary pattern scores at 15 months and nutrient intakes at 18 months were available from *n* = 535 participants. The characteristics of the participants included in the analyses at 6–8 months and at 15–18 months of age were similar (Table 1). Compared with the core ALSPAC cohort, participants included in the present analyses were born to mothers who were slightly older, more likely to be primiparous, have a higher level of education, from social classes II and III, and were more likely to have never smoked or quit smoking. Although the magnitude of these differences is quite small, the statistical analysis was significant at the *p* < 0.01 level due to the large sample size. The proportion of males to females and singletons to twins did not differ from the core cohort.

**Table 1:** Characteristics of the subset of the ALSPAC cohort included in the analysis of patterns and nutrients at 6–8 months or at 15–18 months of age.

		6–8 Months	15–18 Months	Core Cohort
		*n* = 725	*n* = 535	*n* = 13,978 *
**Maternal characteristics**				
Mothers age (year) †		29.3 ± 4.4	29.0 ± 4.4	28.0 ± 5.0
Parity (*n*%)				
	Primiparous	342 (47)	243 (45)	5772 (41)
	Multiparous	369 (51)	287 (54)	7157 (52)
	Missing	14 (2)	5 (1)	1049 (8)
Highest education (*n*%) ‡				
	CSE	77 (11)	48 (9)	2504 (18)
	Vocational	67 (9)	55 (10)	1224 (9)
	O levels	244 (33)	208 (39)	4296 (31)
	A levels	202 (28)	146 (27)	2794 (20)
	Degree	120 (16)	71 (13)	1600 (11)
	Missing	15 (2)	7 (1)	1560 (11)
Mothers social class (*n*%) ‡				
	I	39 (5)	21 (4)	591 (4)
	II	206 (28)	160 (30)	3169 (23)
	III (non-manual)	270 (37)	218 (41)	4304 (31)
	III (manual)	44 (6)	27 (5)	789 (6)
	IV	44 (6)	27 (5)	992 (7)
	V	8 (1)	9 (2)	218 (2)
	Missing	114 (16)	73 (14)	3915 (28)
Financial Difficulties (*n*%) ‡				
	None	639 (88)	479 (89)	10,884 (78)
	Some	45 (6)	33 (6)	1204 (9)
	Missing	41 (6)	23 (4)	1890 (14)
Smoking (*n*%) ‡				
	Never	388 (54)	284 (53)	5804 (42)
	Quit	223 (31)	168 (31)	3457 (25)
	In pregnancy	99 (14)	75 (14)	2173 (16)
	Missing	15 (2)	8 (2)	2544 (18)
Partnered (*n*%) ‡				
	Yes	594 (82)	434 (81)	10,119 (72)
	No	126 (17)	98 (18)	3387 (24)
	Missing	5 (1)	3 (1)	472 (3)
**Infants characteristics**				
Sex (*n*%) ‡				
	Male	389 (53)	299 (56)	7220 (52)
	Female	346 (47)	236 (44)	6756 (48)
Multiplicity (*n*%) ‡				
	Singleton	716 (97)	527 (99)	13,617 (97)
	Twin	9 (1)	8 (1)	361 (3)
Birth weight (kg) †		3.47 ± 0.51	3.46 ± 0.50	3.39 ± 0.51

* Characteristics of the sample at 6–8 months or 15–18 months were compared against the rest of the cohort using chi-squared tests for categorical variables and independent *t*-tests for continuous variables; all characteristics differed between the participants and the core cohort (*p* < 0.001), except for the proportion of girls/boys (*p* = 0.5) and singleton/twins (*p* = 0.1); ^†^ values are reported as mean ± standard deviation; ^‡^ percentages may not add to exactly 100% due to rounding.

### 3.1. Associations between Dietary Patterns at 6 Months and Nutrient Intakes at 8 Months

Table 2 shows the energy and energy-adjusted nutrient intakes at 8 months of age, according to tertiles of dietary pattern scores at 6 months. Scores on the *home-made traditional* pattern were positively associated with median daily energy intake, whereas tertiles of the *ready-preapred baby foods*, *discretionary* and *breastfeeding* patterns showed no significant trend for energy intake. Each dietary pattern had different associations with macro- and micro-nutrients. For example, scores on the *home-made traditional* pattern were positively associated with energy-adjusted intakes of protein, and no association with carbohydrate or fat. Scores on the *discretionary* pattern were not associated with energy-adjusted intakes of protein, carbohydrate or fat. Scores on the *ready-prepared baby foods* pattern were positively associated with carbohydrate. Any possible differences in energy-adjusted intakes of macronutrients across tertiles of the *breastfeeding* pattern were small and not significant. 

**Table 2:** Median daily energy intake and energy-adjusted nutrient intake* at 8 months of age, according to tertiles of dietary pattern scores at 6 months (*n* = 725).

	**Home-made traditional**				**Discretionary**				**Ready-prepared baby food**				**Breastfeeding**			
	T1	T2	T3	*p* ^†^	T1	T2	T3	*p* ^†^	T1	T2	T3	*p* ^†^	T1	T2	T3	*p* ^†^
*n*	242	242	241		242	242	241		242	242	241		242	242	241	
Tot E (MJ)	3.3	3.4	3.5	<0.0005	3.4	3.4	3.4	0.27	3.3	3.4	3.5	0.01	3.4	3.4	3.4	0.08
Protein (g)	25.4	27.4	28.7	<0.0005	27.4	26.6	27.3	0.49	27.8	26.6	27.3	0.11	27.5	27.4	26.5	0.03
Fat (g)	33.3	33.0	33.0	0.90	32.8	33.3	33.2	0.22	33.5	33.8	32.2	0.003	32.8	32.9	33.7	0.11
SFA (g)	14.4	14.3	14.1	0.80	14.0	14.1	14.5	0.04	14.6	14.3	13.6	0.02	14.0	14.1	14.4	0.29
MUFA (g)	11.8	11.7	11.4	0.08	11.5	11.9	11.5	0.41	11.7	11.8	11.4	0.03	11.6	11.5	11.8	0.19
PUFA (g)	4.6	4.5	4.6	0.61	4.6	4.8	4.4	0.04	4.7	4.6	4.5	0.13	4.5	4.6	4.6	0.30
CHO (g)	109	109	106	0.01	108	108	108	0.93	105	108	110	<0.0005	108	108	108	0.80
Starch (g)	36.8	37.9	36.7	0.33	35.9	36.5	38.2	0.02	35.9	36.3	39.0	<0.0005	37.0	35.6	38.8	0.12
Total sugars (g)	71.1	70.3	69.5	0.03	70.9	70.9	68.5	0.01	70.9	70.4	69.7	0.37	70.8	72.0	68.5	0.06
NSP (g)	3.3	4.2	4.7	<0.0005	4.2	4.1	3.9	0.01	4.5	3.9	3.8	<0.0005	3.8	3.8	4.5	<0.0005
Sodium (mg)	532	546	644	<0.0005	544	551	615	<0.0005	598	543	559	0.02	573	549	591	0.60
Potassium (mg)	1.1	1.2	1.3	<0.0005	1.2	1.2	1.2	0.21	1.2	1.2	1.2	0.17	1.2	1.2	1.2	0.68
Calcium (mg)	662	615	598	<0.0005	623	613	645	0.25	584	634	660	<0.0005	650	650	578	<0.0005
Iron (mg)	8.7	8.2	7.1	<0.0005	7.9	8.1	8.2	0.78	7.1	8.1	9.0	<0.0005	8.2	8.5	6.9	<0.0005
Zinc (mg)	4.5	4.5	4.5	0.65	4.6	4.6	4.4	<0.0005	4.4	4.5	4.5	0.38	4.6	4.6	4.2	<0.0005
Phosph (mg)	580	586	596	0.41	593	575	599	0.27	577	585	597	0.01	614	597	556	<0.0005
Magnesium (mg)	98	103	109	<0.0005	104	102	105	0.68	104	103	102	0.14	103	103	104	0.61
Copper (mg)	0.60	0.60	0.54	<0.0005	0.60	0.60	0.56	<0.0005	0.54	0.58	0.61	<0.0005	0.58	0.58	0.57	0.80
Manganese (mg)	0.93	0.92	0.97	0.01	0.97	0.95	0.92	0.32	0.96	0.92	0.98	0.77	0.91	0.91	1.00	0.002
Selenium (µg)	11.8	13.6	17.7	<0.0005	13.4	13.8	15.4	0.03	16.2	13.5	12.7	<0.0005	12.8	13.1	16.5	<0.0005
Iodine (µg)	75	78	80	0.02	78	78	77	0.79	78	77	79	0.62	76	78	78	0.64
Thiamine (mg)	0.95	0.90	0.81	<0.0005	0.89	0.89	0.88	0.95	0.77	0.91	0.97	<0.0005	0.92	0.90	0.80	<0.0005
Riboflavin (mg)	1.20	1.19	1.17	0.56	1.17	1.18	1.21	0.06	1.14	1.19	1.23	0.001	1.23	1.23	1.05	<0.0005
Niacin Eq (mg)	12.8	13.3	13.7	<0.0005	13.7	13.3	13.0	0.01	13.4	13.1	13.3	0.55	13.4	13.5	12.8	0.04
Folate (µg)	95	99	104	<0.0005	103	99	95	<0.0005	100	99	99	0.22	99	99	99	0.75
Vit. B 6 (mg)	0.65	0.72	0.80	<0.0005	0.75	0.72	0.70	0.004	0.73	0.72	0.71	0.005	0.72	0.74	0.70	0.12
Vit. B12 (µg)	1.9	1.9	1.9	0.19	1.9	1.9	2.0	0.28	1.9	1.9	1.9	0.84	2.0	2.0	1.7	<0.0005
Vit. C (mg)	84	77	70	<0.0005	82	77	74	0.012	66	81	86	<0.0005	84	85	67	<0.0005
Vit. D (µg)	5.8	5.3	4.8	<0.0005	5.4	5.7	4.5	0.005	4.8	5.3	5.6	0.02	5.7	6.3	2.4	<0.0005
Vit. E (mg)	5.5	5.3	4.8	<0.0005	5.3	5.3	4.9	0.002	5.0	5.2	5.6	0.003	5.5	5.5	4.6	<0.0005
Retinol Eq (µg)	902	863	802	<0.0005	887	875	775	<0.0005	820	870	893	0.006	868	937	770	0.001

CHO, carbohydrate; Eq, equivalents; MUFA, monounsaturated fatty acid; PUFA, polyunsaturated fatty acid; NSP, non-starch polysaccharide; phosph, phosphorous; SFA, saturated fats; T, tertile; Tot E, total energy; vit, vitamin; * energy-adjusted nutrient intake was calculated using the residual method [28]; ^†^ Cuzick’s non-parametric test for trend across ordered groups (which is based on the Wilcoxon rank-sum test but extended to compare more than two groups) was used to determine whether median nutrient intakes differed across tertiles of pattern scores [27], with a Bonferroni-adjusted *p*-value of <0.0005 .

For transparency we have presented an extensive list of micronutrients in Table 2, but here we draw attention to dietary components of public health importance that are linked to healthy child development. The profile of sodium and potassium intakes differed for each dietary pattern. Scores on the *breastfeeding* pattern were not associated with sodium or potassium intake, while the difference in median sodium intake for the *discretionary* pattern was approximately 70 mg. However, the *home-made traditional* pattern was positively associated with both sodium and potassium intake, and of all the patterns, had the widest difference in sodium intake from the highest to lowest tertile (~100 mg). Calcium and iron intakes decreased across tertiles of the *home-made traditional* and *breastfeeding* patterns, by ~70 mg and ~1.5 mg, respectively. However, calcium and iron intake increased across tertiles of the *ready-prepared baby food* pattern scores by 70 mg and 1.9 mg. NSP intake increased across tertiles of the *home-made traditional* and *breastfeeding* patterns, while a decreasing association was observed for the *ready-prepared baby foods* pattern. 

### 3.2. Associations between Dietary Patterns at 15 Months and Nutrient Intakes at 18 Months

Table 3 shows the total energy and energy-adjusted nutrient intakes at 18 months of age, across tertiles of dietary pattern scores at 15 months. Total energy intakes were quite stable across tertiles of all dietary patterns. Of the patterns extracted at 15 months of age, the *home-made contemporary* pattern is likely to be the healthiest because we have previously shown that high scores were associated with higher intakes of fruit, vegetables, legumes and cheese [7]. With this in mind, higher scores on the *home-made contemporary* pattern at 15 months of age were associated with 1.6 g lower energy-adjusted intakes of saturated fat between the lowest and highest tertiles, and 0.9 g higher NSP intakes, but no association with other macronutrients.

**Table 3:** Median energy and energy-adjusted nutrient intake * at 18 months of age, according to tertiles of dietary pattern scores at 15 months (*n* = 535).

	Home-Made Contemporary				Discretionary				Ready-Prepared Baby Foods				Home-Made Traditional			
	T1	T2	T3	*p* ^†^	T1	T2	T3	*p* ^†^	T1	T2	T3	*p* ^†^	T1	T2	T3	*p* ^†^
*n*	179	178	178		179	178	178		179	178	178		179	178	178	
Tot E (MJ)	4.4	4.5	4.5	0.23	4.5	4.5	4.6	0.05	4.4	4.5	4.6	0.06	4.5	4.6	4.5	0.52
Protein (g)	40.9	41.2	41.4	0.44	41.9	41.3	40.1	0.001	42.1	41.0	40.7	0.12	40.1	40.9	42.6	<0.001
Fat (g)	48.0	47.1	46.6	0.006	47.3	46.9	47.0	0.71	47.7	46.8	46.8	0.08	47.0	47.2	47.1	0.69
SFA (g)	23.4	23.1	21.8	<0.0005	22.8	22.8	22.4	0.90	23.0	22.2	22.7	0.63	21.8	23.2	22.8	0.03
MUFA (g)	15.2	14.7	14.9	0.007	15.0	15.0	15.0	0.51	15.0	15.0	14.9	0.34	15.0	14.8	15.0	0.89
PUFA (g)	4.7	4.8	5.4	0.002	4.9	5.1	5.0	0.89	4.9	5.2	4.7	0.11	5.1	5.1	4.7	0.04
CHO (g)	134	137	137	0.07	135	136	137	0.18	133	136	137	0.03	139	134	136	0.03
Starch (g)	59.9	59.9	58.7	0.35	57.3	61.6	60.1	0.05	60.0	59.9	58.8	0.32	61.3	58.1	59.6	0.17
Total sugars (g)	71.7	72.8	76.9	0.002	74.4	73.5	74.0	0.67	71.7	73.6	76.7	0.004	74.6	72.9	73.5	0.52
NSP (g)	6.0	6.3	6.9	<0.0005	6.8	6.2	6.2	0.01	6.3	6.6	6.3	0.69	6.4	6.3	6.6	0.99
Sodium (g)	1.38	1.43	1.36	0.89	1.38	1.40	1.42	0.07	1.45	1.41	1.35	0.001	1.39	1.41	1.38	0.97
Potassium (g)	1.68	1.71	1.80	<0.005	1.78	1.70	1.71	0.01	1.69	1.71	1.63	0.19	1.72	1.69	1.46	0.15
Calcium (mg)	825	820	758	0.03	834	785	779	0.005	814	783	812	0.80	764	810	817	0.02
Iron (mg)	4.9	4.9	5.2	0.07	5.3	5.0	4.9	0.003	4.8	5.2	5.1	0.003	5.1	4.9	5.0	0.07
Zinc (mg)	4.9	4.8	4.8	0.70	5.1	4.8	4.6	<0.0005	4.9	4.8	4.8	0.97	4.8	4.8	4.9	0.07
Phosphorous (mg)	890	873	873	0.77	900	876	860	<0.0005	884	869	886	0.87	866	871	901	0.002
Magnesium (mg)	146	150	158	<0.0005	157	151	144	<0.0005	149	151	152	0.58	152	149	152	0.50
Copper (mg)	0.39	0.41	0.46	<0.0005	0.43	0.43	0.41	0.07	0.41	0.41	0.45	0.002	0.44	0.42	0.41	0.03
Manganese (mg)	1.17	1.17	1.36	<0.0005	1.36	1.19	1.18	0.004	1.21	1.30	1.18	0.37	1.30	1.24	1.17	0.23
Selenium (µg)	31.1	32.9	34.3	<0.0005	34.0	33.4	31.9	0.03	33.7	33.6	30.9	0.03	33.3	33.1	32.7	0.92
Iodine (µg)	176	172	171	0.85	182	151	176	0.22	186	169	163	0.19	143	176	188	0.01
Thiamine (mg)	0.82	0.84	0.85	0.43	0.87	0.82	0.82	0.001	0.82	0.83	0.87	0.01	0.83	0.82	0.86	0.17
Riboflavin (mg)	1.54	1.50	1.44	0.15	1.55	1.49	1.44	0.003	1.51	1.44	1.52	0.75	1.42	1.50	1.55	0.003
Niacin Eq (mg)	16.1	16.7	17.0	0.01	17.0	16.7	16.4	0.003	16.8	16.7	16.2	0.04	16.5	16.5	17.1	0.009
Folate (µg)	124	126	131	0.001	132	125	123	0.001	125	130	127	0.75	125	127	129	0.54
Vit. B 6 (mg)	1.06	1.06	1.12	0.08	1.15	1.03	1.07	0.02	1.08	1.12	1.05	0.21	1.07	1.07	1.09	0.34
Vit. B12 (µg)	3.1	3.2	3.1	0.53	3.2	3.1	3.1	0.08	3.2	3.2	3.1	0.50	2.9	3.2	3.2	0.01
Vit. C (mg)	32	36	47	<0.0005	44	39	34	0.003	32	42	44	<0.0005	41	35	39	0.44
Vit. D (µg)	1.15	1.16	1.91	0.43	1.20	1.16	1.15	0.08	1.19	1.15	1.11	0.14	1.21	1.25	1.06	0.001
Vit. E (mg)	4.03	3.96	4.37	0.06	4.05	4.07	4.14	0.36	4.06	4.19	4.00	0.78	4.37	4.06	3.86	0.004
Retinol Eq (µg)	611	566	554	0.02	597	578	545	0.03	540	560	624	0.001	589	577	571	0.95

CHO, carbohydrate; Eq, equivalents; MUFA, monounsaturated fatty acid; PUFA, polyunsaturated fatty acid; NSP, non-starch polysaccharide; phosph, phosphorous; SFA, saturated fats; T, tertile; Tot E, total energy; vit., vitamin; * energy-adjusted nutrient intake was calculated using the residual method [28]; ^†^ Cuzick’s non-parametric test for trend across ordered groups (which is based on the Wilcoxon rank-sum test but extended to compare more than two groups) was used to determine whether median nutrient intakes differed across tertiles of pattern scores [27], with a Bonferroni-adjusted *p*-value of <0.0005.

The association between patterns and energy-adjusted intakes of sodium and potassium were weak. The difference in energy-adjusted sodium intake between the highest and lowest tertiles of all patterns was only 0.1 g per MJ, indicating that the magnitude of the difference is small. Similarly, median iron intakes differed by a maximum of only 0.3 mg in the lowest compared with the highest tertiles of all patterns. Higher scores on the *discretionary* pattern were associated with lower intakes of zinc, phosphorous and magnesium, and higher *home-made contemporary* pattern scores were positively associated with magnesium, copper, manganese and selenium, although the magnitude of the differences in nutrient intakes between the highest and lowest tertiles is small.

## 4. Discussion

The present study shows that the dietary patterns of infant and toddlers exhibit variation in their underlying energy and nutrient composition. Each pattern was associated with a different profile of nutrients. We have previously shown that the intake of foods vary across quartiles of pattern scores at 6 and 15 months of age [7]. The trends in nutrient intakes across dietary pattern scores presented here further demonstrate that the patterns obtained using questionnaires describe dietary differences not only in food intakes but also in nutrient profile.

Sodium intake tended to increase while many other nutrients tended to decrease across tertile of *discretionary* pattern scores. These results suggest that higher scores on the *discretionary* pattern reflect a poorer nutritional profile and a less nutrient dense diet. In contrast, the *ready-prepared baby foods* pattern was positively associated with key micronutrients such as iron and calcium, yet negatively associated with sodium intake. However scores on the *ready-prepared baby foods* pattern was also negatively associated with NSP intake at 6–8 months. These results suggest a generally positive nutritional profile with some room for improvement. The favorable nutrient composition of the *ready-prepared baby foods* pattern is pertinent given than the UK infant feeding guidelines specifically promote the use of home-made rather than ready-prepared baby foods [29].

The associations between the *home-made traditional* dietary patterns with energy and nutrient intake were more difficult to interpret and showed little consistency between 6–8 and 15–18 months of age. At 6–8 months, higher scores on the *home-made traditional* pattern were positively associated with energy, protein, NSP and sodium intake, and negatively associated with calcium and iron. However at 15–18 months of age, the association between the *home-made traditional* pattern and protein intake was consistent with the data at 6–8 months, but there was no association with carbohydrate, calcium, sodium and iron intake. These results suggest the traditional patterns at different ages reflect a mixed nutrient profile, making it difficult to hypothesize how the traditionalpatterns might influence health and development. There was limited variation in fat intake across the dietary patterns at either time point, which may limit their use in predicting outcomes such as blood lipids.

At 6–8 months of age, the *breastfeeding* pattern was negatively associated with calcium and iron intake. It is important to note that breast milk intake was not assessed directly, instead mothers reported the duration of each breastfeed and an algorithm was applied to the rate of transfer of milk to the infant [30]. Furthermore, the nutrient content of breast milk does not allow for the bioavailability of the nutrient, which is often enhanced in breast milk. Therefore, the associations between scores on the *breastfeeding* pattern and nutrients may be less reliable than for other patterns. Nevertheless, median nutrient intakes exceeded the UK reference values [31] suggesting that the predominantly breastfeeding pattern reflects an age-appropriate nutritional profile. Finally, the *home-made contemporary* pattern at 15–18 months of age was negatively associated with intakes of saturated fat, and positively with NSP, potassium and iron. With respect to the macronutrient intakes, higher scores on the *home-made contemporary* pattern tended to reflect a healthier diet.

The present study is one of the few that have examined the association between dietary patterns and nutrient intakes in a pediatric sample [32]. Consistent with previous literature [11,12], we found that patterns characterized by higher intakes of healthier foods had more positive nutrient profiles than patterns characterized by higher intake of discretionary (nutrient-poor) foods. Even though the present study was conducted in a subset of a large cohort, the number of children involved is substantial (*n* = 535–725) and to the best of our knowledge, this is the only study to have examined associations between nutrient intakes obtained from diet diaries and dietary patterns in the first year of life. 

The present findings should be interpreted within the context of the study strengths and limitations. The ALSPAC cohort is one of the most well-characterized birth cohorts in the world and is considered broadly representative of the population living in the local area during the early 1990’s when the study commenced. Although the dietary data was collected prospectively, the questionnaires were not validated in the study population. While the questionnaire was adapted to the age of the infant, all studies that involve dietary questionnaires have the potential for recall bias. The fact that the dietary patterns were not extracted at the same age as the nutrient data may increase the measurement error. We have assumed that the patterns at 6 and 15 months of age would be similar to those at 8 and 18 months, respectively. This is because new foods are usually introduced to an established repertoire of foods and therefore we may expect to see minor or gradual changes over time, rather than a major change to a different style of diet. Furthermore, the effect of measurement error is likely to result in an underestimation or null association between dietary patterns and nutrient intakes rather than an over-estimation. This may have also contributed to some of the inconsistencies in the associations across each age. By contrast, a strength of this analysis is that associations are observed using two independent measures of diet (*i.e.*, dietary patterns derived from questionnaires and nutrient intakes from diet diaries), because the results may be less influenced by the disadvantages or reliance on one particular method of dietary assessment.

An advantage of using PCA to summarize the whole diet is that the dietary patterns reflect what individuals actually eat. However as dietary patterns are based on actual consumption patterns rather than an established diet-disease relationship, patterns may or may not be associated with health outcomes [33]. The present article highlights the complexity of the associations between dietary patterns and nutrient intakes, as pattern scores may be associated with nutrients that are frequently linked to higher risk of disease (e.g., sodium, saturated fat) and also to protective factors (e.g., iron, NSP). Our analysis demonstrates that an understanding of the nutrient intakes underpinning a dietary pattern can provide important and in insightful information that may be helpful for interpreting the association between dietary patterns and health.

## 5. Conclusions

Early life dietary patterns vary in their underlying energy and nutrient composition. By gaining insight into the energy and nutrient intakes underpinning each pattern, our overall understanding of dietary patterns is improved. While pattern food loadings suggest that the *discretionary*, *ready-prepared baby foods*, and *home-made traditional* patterns are similar at 6 and 15 months of age, the nutrient analysis show that these patterns exhibit some differences in their associations with nutrient intakes.
